# Is the relationship between parental abuse and mobile phone dependency (MPD) contingent across neighborhood characteristics? A multilevel analysis of Korean Children and Youth Panel Survey

**DOI:** 10.1371/journal.pone.0196824

**Published:** 2018-05-03

**Authors:** Harris Hyun-soo Kim, JongSerl Chun

**Affiliations:** 1 Department of Sociology, Ewha Womans University, Seoul, Republic of Korea; 2 Department of Social Welfare, Ewha Womans University, Seoul, Republic of Korea; University of Kansas, UNITED STATES

## Abstract

Research indicates that mobile phone dependency (MPD) is associated with various behavioral and internalizing problems. While a significant amount of findings points to its negative outcomes, there is a dearth of evidence concerning the determinants of MPD. This study focuses on this critical, yet understudied, subject by analyzing the associations between abusive parenting style, neighborhood characteristics, and MPD among youths in South Korea, a country with one of the highest mobile broadband penetration rates in the world. Based on the secondary analysis of two waves of Korean Children and Youth Panel Survey (KCYPS), a government-funded multiyear study, we investigate individual- and contextual-level factors underlying MPD. Findings show that, net of a host of time-lagged controls (including baseline dependency from the previous year), abusive parenting style significantly increases adolescent MPD. After adjusting for individual level characteristics, however, no contextual-level effect is found, i.e., residing in a neighborhood with a relatively higher proportion of parental abuse is not related to greater MPD. Finally, two cross-level interaction effects are observed. First, the association between parental abuse and MPD is weaker in a neighborhood context with better educated inhabitants (more college graduates). Second, it is reinforced in demographically “aged” communities with more elderly residents.

## Introduction

A World Bank report indicates that smartphone ownership has spread rapidly throughout the globe, with the latest average cellular subscription rate at 96.8 per 100 individuals [1]. Even among emerging and developing nations, the median ownership has risen at an extraordinary pace from 21% in 2013 to 37% just two years later [2]. With growing popularity and given the vast amount of time people spend on their mobile phones, excessive usage has emerged as a widespread behavioral addiction [3,4,5]. And the problematic use of mobile phones has been linked with a variety of adverse psychological and behavioral outcomes in daily life [6]. Even new medical terminologies have emerged such as “Facebook Depression” especially among children and adolescents [7]. In light of the growing online overdependence and its problems, the American Psychiatric Association decided to include ‘Internet use disorder’ in the appendix of the fifth edition of the *Diagnostic Statistical Manual for Mental Disorders* [8].

According to the latest data released by Organization for Economic Co-operation and Development, South Korea, along with eleven other countries, lies above the 100% penetration threshold in terms of wireless mobile subscription rate [9]. With widely available access to high-speed Internet connection, the mobile phone dependency (MPD) among Korean youths, in particular, has become a major public health concern. In developing the construct of MPD, scholars have proposed various Smartphone dependency scales, including the modified version of the Kimberly Young Internet addiction test. A study based on Korean adults shows alternative MPD scales to have high internal consistency and validity [10]. A national survey conducted by the Korean Ministry of Gender Equality and Family estimates the mobile phone ownership rate among adolescents at 91.6% [11]. The study also states that 17.3% of the young respondents often felt anxious when not in possession of their mobile devices, while 6.2% of them have experienced the so-called “phantom vibration syndrome.” Overall, as the report concludes, approximately 15% of Korean young users exhibited signs of MPD. A more recent government study finds 25.5% of Korean children (compared to 8.9% of adults) to be addicted to mobile phone use [12]. In response, academics and policy makers alike have sought to gauge and monitor the behaviors of problematic users [10,13]. Under project titles such as “Development of a Korean Smartphone Addiction Proneness Scale for Youths and Adults” [14], massive government efforts have been under way to curb what many see as a growing social ill.

Past research suggests that MPD among young users is associated with a host of internalizing problems, including depression [15,16,17] and suicidal ideation [18,19], as well as physical health symptoms such as headaches [20], tinnitus [20], insomnia and sleep disturbance [21,22], and neck and shoulder pain [23]. Furthermore, suspension from school [21] and financial problems [24] were also found to be related to adolescent MPD. While a significant amount of evidence has accumulated documenting its consequences, much less research has been devoted to analyzing the antecedents of MPD. The limited research on the topic narrowly focus on personal and psychological characteristics [4]. For example, gender [25], depression [25], low self-esteem [21], stress and loneliness [12], aggression [26], school adjustment [12,25], delinquency [21,25,27], and family income [25] have all been cited as possible factors related to problematic use of mobile phone.

While fully recognizing the contributions of earlier research, we seek to contribute to the extant scholarship by moving beyond connecting individual attributes (gender, depression, loneliness, delinquency, etc.) with adolescent MPD. Specifically, our aim is to investigate the complex interplay between parenting style (quality of parent-child relationship), neighborhood characteristics, and dependency on mobile phone among Korean youths. Among other purposes, mobile devices are primarily intended and used for interpersonal communication [28]. Hence, it is critical to consider the relational context in which MPD gets formed. In childhood and adolescence, one’s primary relationship is with parents [29]. As a result, parental abuse, first and foremost, has been recognized as a powerful predictor of children’s dysfunctional development [30]. Parental abuse is shown to foster coercive social relations and antisocial behaviors among children [31]. The short-term and long-term effects of parental abuse also include a wide range of physical, psychological and behavioral problems such as neurological and musculoskeletal problems, depression, anxiety, substance abuse, suicide attempts, and risky sexual behaviors in both childhood and adulthood [32,33].

A limited number of studies have examined the influence of abusive parenting style on Korean children’s MCD, albeit with disparate results [29,34,35]. Despite the lack of consensus on the exact relationship between the two, it is generally argued that a child with abusive parents is more likely to experience MPD. Two significant limitations, however, are found in these studies. First, they are all based on cross-sectional data. Consequently, the direction of causality between parental abuse and MPD cannot be established. The current study addresses this problem by using two waves of longitudinal, nationally representative data on Korean school-aged children. By doing so, we minimize, though not resolve, the issue of endogeneity and offer more conclusive evidence on the causal linkage between the two variables. Second, prior research measures parental abuse only at the individual (e.g., child) level. Given this methodological limitation, it is not possible to probe independent and interactive or moderating effects of contextual-level variables (neighborhood characteristics), which have been known to affect adolescent mental and physical well-being [36,37,38]. Does aggregate-level parental abuse at the neighborhood or community level have an impact on a child’s MPD, irrespective of whether or not s/he experiences parental abuse at home? And to what extent do residential characteristics moderate the association between parental abuse and adolescent MPD? These are conceptually and empirically pertinent questions that, for the most part, have not received systematic scholarly attention.

According to the oft-cited ecological model [39], all human behaviors develop as a result of following three factors: (1) the individual’s awareness of the environment, (2) the individual’s environment, and (3) the interaction between the individual and the environment. In line with this theoretical framework, an increasing number of studies have examined how neighborhood-level contextual factors impact various externalized behaviors and internalizing problems of children and adolescents [40,41,42,43,44,45,46]. The main question is whether and to what extent broader neighborhood characteristics are associated with differential health outcomes, net of individual-level factors or adjusting for compositional effects. In the epidemiological literature, the concept of collective efficacy has been frequently operationalized to gauge the levels of social capital and potential for collective action among neighbors in relation to health inequality [47]. Studies have shown that a neighborhood with greater collective efficacy is more protective against stressors that undermine physical and mental health, for adults as well as youths [38, 48]. Another critical factor is the neighborhood socioeconomic status, i.e., average household income, which has been positively associated with the wellbeing of residents of all ages, including children and adolescents [43]. Research indicates, for example, that there is a significant relationship between living in high-poverty neighborhoods, which is associated with limited community social support, and greater youth internalizing symptoms [49].

Concerning the health and health related behaviors of youth, family environment also plays a critical role [50]. Prior studies show that family structure (single-parent versus dual-parent) and other familial factors are related, specifically, to Korean youths’ smartphone addiction [51,52,53]. Demographically, Korea has undergone a major transformation during the last few decades. The country has one of the highest divorce rates among OECD nations [54] and is considered the fastest ageing society in the world today [55]. At 69%, compared to the average of 42%, Korea also boasts of the highest proportion of people in their 20s and 30s with a college degree among OECD nations [56]. We conjecture that these demographic trends and facts related to family makeup, age structure, and educational level constitute key dimensions of neighborhood environment with consequential implications for MPD among young Koreans. According to extant literature, the prevalence of adolescent mental disorders varies significantly across, along with household income, parental education and parenthood structure [57]. Such features together form an ecological context that shapes children’s growth and development [58]. In fact, one of the consistent findings is that residence in a deprived neighborhood defined in terms of lower aggregate family socioeconomic status (SES), i.e., living in areas with less educated parents with limited earnings, is related to poor emotional and behavioral outcomes [43]. Another critical but often neglected issue is the neighborhood age structure (e.g., proportion of older residents), which correlates highly with the level of community infrastructure and availability of community resources [59]. The age composition of neighborhood also implies different kinds of social interaction that affect the daily lives of individuals [60]. In Korea, given the rapid aging process, “older” communities have been increasingly found to be associated with higher rates of poverty [61,62] and lower quality of living [63]. In fact, it is widely recognized that the proportion of elderly residents varies inversely with economic productivity and local development [64].

**Fig 1:**
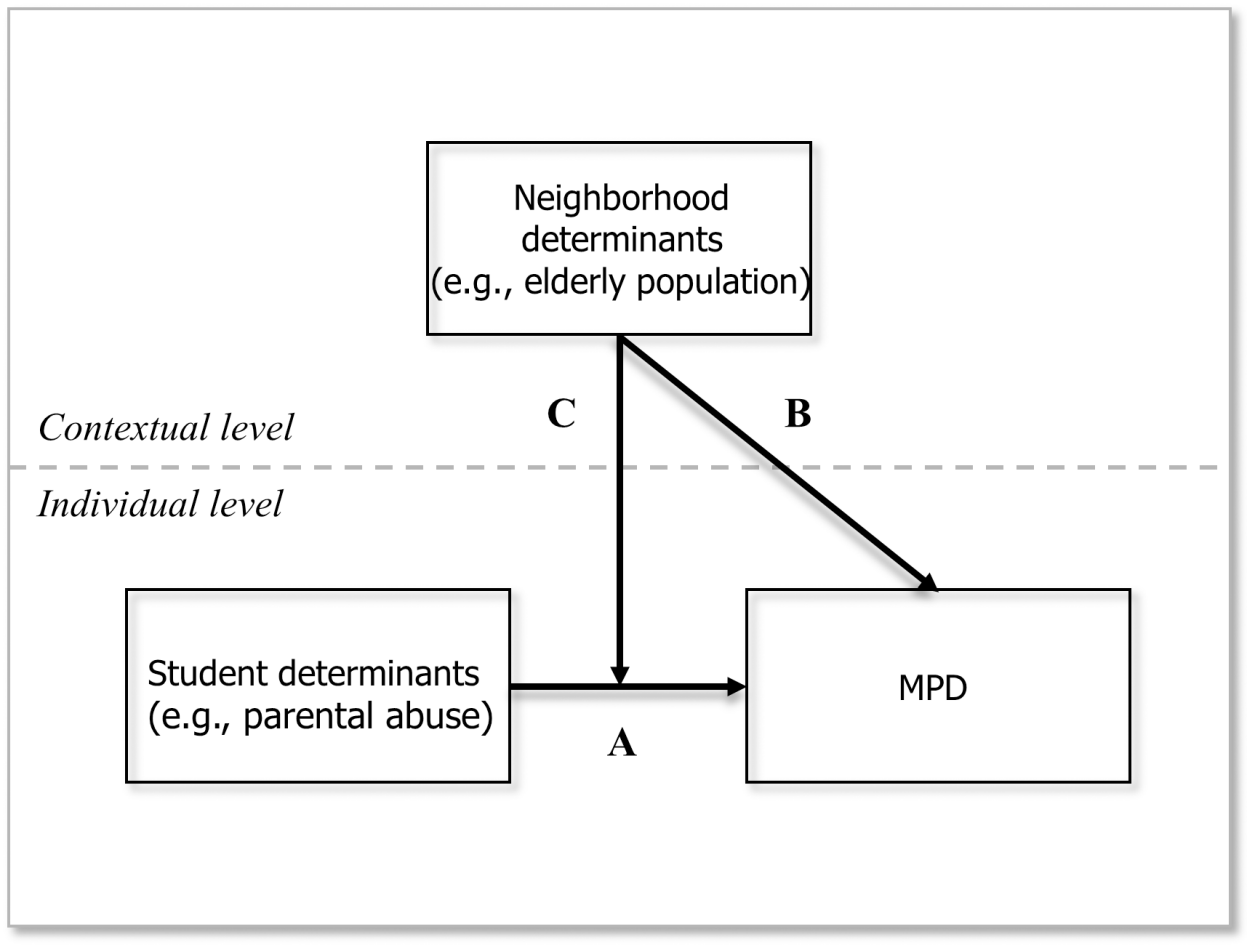
Multilevel explanatory framework.

In light of previous findings and Korea’s demographic characteristics, we take into account the following three contextual-level variables that serve as proxies for neighborhood quality: divorce rate, proportion of elderly residents, and average level of educational attainment. Neighborhoods that vary along such dimensions differ in their community resources and capacity that can have an impact on individual-level outcomes, especially with respect to youths [38, 49]. On the one hand, a residential context with more intact (two-parent) families, lower percentage of the elderly population, and better-educated residents can provide a more effective stress-buffering mechanism for younger residents. Neighborhood characteristics, on the other hand, can also have a more indirect effect by moderating the relationship between an outcome (e.g., cellphone dependency) and a predictor (e.g., parenting style). In this study, we hypothesize that adolescent MPD is a function of both individual- and environmental-level variables. More specifically, we test the relationship between MPD and parental abuse (arrow A) and the possibility that it is independently (arrow B) and interactively (arrow C) affected by neighborhood characteristics, as illustrated by the diagram in Fig 1.

Like any other behavioral problems, adolescent MPD should be understood from an ecological perspective or within a multilevel framework. However, only a handful of studies (published in Korean) have explicitly considered the role of broader environmental issues in shaping MPD among young users [65, 66, 67]. And, to our knowledge, no study has probed cross-level interactions between individual- and contextual-level covariates. The main objective of our study, therefore, is to fill this research gap by using hierarchical linear models to explore the complex ways in which adolescent MPD is shaped by abusive parenting style and neighborhood qualities, and also discuss implications of the empirical findings that extend beyond the Korean context.

## Materials and methods

### Data source

Data are drawn from the Korean Children and Youth Panel Survey (KCYPS), a series of longitudinal data collection efforts funded by the Korean government (Ministry of Gender Equality and Family) and carried out by the National Youth Policy Institute (NYPI), the largest public think tank of its kind in the country. The research protocol was approved by the Institutional Review Board associated with the National Institute of Youth Studies, Korea. Findings reported below are from the secondary analysis of two sets of data collected in 2012 (Wave 3) and 2013 (Wave 4). Based on the multiple point prospective panel design, a probability sample (N = 2,351) representing all middle school students in Korea was chosen and surveyed every year since 2010. The Wave 4 sample consisting of first-year high school students has the retention rate of 89.7%. Due to the bias stemming from the sample attrition, individual weights were applied to adjust for the data representativeness. Stratified multi-stage cluster sampling was used to first select a group of schools throughout the country. Out of the total population of 2,320 schools, 78 were chosen using the probability proportion to size (PPS) sampling method. For each school, one specific class was selected and all students present in the classroom filled out the survey questionnaire. After excluding cases with item-level missing data, the effective sample consists of 1,788 respondents (51% boys) with valid sample weights who were nested in 85 residential communities (geographically demarcated administrative units akin to census tracts in the US but on average larger in population size). Further information about the data and sampling strategies can be found at the data archive homepage of NYPI [68].

### Measures

To establish proper temporal (causal) order between the variables of interest, we construct the outcome variable (*Mobile phone dependency*) from Wave 4 (2013) while all other time-lagged variables are taken from Wave 3 (2012). This variable was measured using the cell phone overuse scale developed by Lee et al. (2002), one of the most frequently used instruments for MPD among Korean children and adolescents. An abridged version of alternative scales available (Kwon et al. 2013), it consists of 7 items using a 5-point Likert scale, with higher scores indicating higher levels of MPD. The KCYPS questionnaire contains these items which were specifically designed to tap the respondents’ levels of dependency (e.g., “I feel anxious without my mobile phone,” “I feel insecure if I do not receive a call from someone,” “I feel lonely and socially disconnected without my phone”). Individual answers were averaged to create a composite score for MPD. The main predictor variable is *Parental abuse*. The survey questionnaire contains multiple items that explicitly probe the quality of parent-child relationship. The student respondents were inquired about the ways in which they had been treated by their parents on a daily basis. Among the questions are those that measure abusive parenting style (e.g., “My parents severely punish me if I do something even slightly wrong,” “I have often been verbally abused by them”). Answers were summed across 4 items and averaged, with a higher value indicating greater abuse.

In empirically investigating the associations between parental abuse and MPD among Korean youths, we incorporate a number of control variables at both individual and contextual levels. First and foremost, at the individual level, we take advantage of the longitudinal design of KCYPS by adjusting for baseline dependency from the earlier wave. The inclusion of the variable, *(W3) MPD*, allows for a more conservative test of the potential effect of abusive parenting style on children’s dependency. We also take into account key sociodemographic background factors including gender, household income and family arrangement (whether the respondent lives with both biological parents). Subjective assessment of one’s academic achievement is further included, along with the child’s level of social interaction (how often s/he interacts with friends). Three behavioral and psychological measures are taken into account as well. First, a composite index of delinquent behaviors (smoking, drinking, bullying, gambling, etc.) is constructed. Second, answers to several items are compiled to measure aggressiveness (e.g., “If I can’t get things my way, I resort to being argumentative or violent”). Lastly, a depression scale is included based on 10 items modelled after the Center for Epidemiological Studies Depression Revised (CESD-R). Cronbach’s alpha values for all the composite scores show acceptable levels of internal validity.

In addition to these individual-level variables, several neighborhood characteristics are adjusted for to test independent and moderating contextual-level effects. Extant studies deal with a variety of contexts that shape youth behaviors, such as peer relations, family and school. “Neighborhood effects,” on the other hand, have not received much analytic attention. This study thus focuses on three residential community characteristics related to Korea’s unique demographic trends- aggregate divorce rate, average educational attainment, and proportion of elderly inhabitants- by drawing on official government data available from the Korean Statistical Information Service (KOSIS) [69]. Geocoding was based on the respondents’ addresses provided during the fourth wave of data collection. Availability of this information allowed us to gather KOSIS aggregate statistics and merge them with the (respondent-level) KCYPS data. Since neighborhood poverty level influences adolescent development [43,46,49], average household income is also included by calculating the mean value for each neighborhood unit based on the original answers provided by respondents. Based on individual answers to a host of questions, we also created an index that measures “collective efficacy,” or community social capital, a well-established concept in the literature that has been shown to influence adolescent mental and physical development [38,44,48]. Finally, an aggregate measure is constructed to examine if and to what extent being in a residential neighborhood characterized by higher average parental abuse shapes adolescent MPD, over and above the individual-level effect. Table 1 summarizes the survey items and coding scheme used for variable construction. Basic descriptive statistics are provided in Table 2.

**Table 1:** Summary of variable definitions and coding scheme.

Outcome measure	
*Mobile phone dependency*	“I find myself spending an increasing amount of time on my mobile phone”; “I feel anxious without my phone”; “I feel insecure if I do not receive a call from someone”; “I lose track of time while using my phone”; “I feel extremely bored without my phone when I’m alone”; “I feel lonely and socially disconnected without my phone”; “I feel extremely inconvenient without my phone.” Individual answers from Wave 4 averaged to create a composite score (Cronbach’s alpha = .88)
Level-1 covariates (N = 1,788)	
*Female*	Respondent’s (R’s) gender; coded 1 if female.
*Academic satisfaction*	R’s overall satisfaction with his/her academic achievement (4 = “very satisfied,” 3 = “satisfied,” 2 = “not satisfied,” 1 = “very dissatisfied”).
*Delinquency*	An index based on answers (coded 1 if “yes”) to 6 delinquent behaviors, including smoking, drinking, truancy, running away from home, bullying, and gambling (alpha = .58).
*Aggressiveness*	“I tend to get annoyed at minor things”; “I interfere with other people’s business”; “If I can’t get things my way, I resort to being argumentative or violent”; “I fight over trivial matters”; “Sometimes I stay angry all day”; “I can get very upset and depressed for no apparent reason” (4 = “all the time,” 3 = “most of the time”; 2 = “sometimes”; 1 = “rarely”). Responses were averaged and log-transformed due to skewed distribution (alpha = .78)
*Social interaction*	Logged number of hours spent socializing with friends during the week.
*Household income*	Annual household income (ln)
*Both parents*	Coded 1 if R lives with both biological parents
*Depression*	A scale based on 10 items modelled after the Center for Epidemiological Studies Depression Revised (CESD-R). Individual answers were averaged (Cronbach’s alpha = .89) and then log-transformed due to right-tailed skewed distribution.
*(W3) MPD*	A composite score based on 7 items regarding cell phone dependency taken from the previous year, i.e., Wave 3 (alpha = .90).
*Parental abuse*	“My parents severely punish me if I do something even slightly wrong”; “My parents try to physically hit me whenever they find fault with me”; “I have received bruises on my body from them”; “I have often been verbally abused by them” (alpha = .85). Averaged responses to these survey items.
Level-2 covariates (N = 85)	
*Collective efficacy*	Averaged individual answers to the following statements: “I know most of my neighbors on a personal basis”; “I greet my neighbors if I see them on the street”; “My neighbors generally get along with each other”; “I believe my neighborhood is safe”; “I enjoy spending time with my neighbors”; “I wish to continue to live in my neighborhood” (alpha = .70).
*Aged population*	Calculated as an “ageing index” (ratio of the number of elderly over the age of 65 to those under the age of 15 multiplied by 100)
*Divorce rate*	Proportion of divorced families
*College educated*	Percentage of residents who graduated from college
*Average income*	Average household income (ln)
*L2_Parental abuse*	Community-level mean value for parental abuse, as defined above

*Data source*: KCYPS (2012 & 2013)

**Table 2:** Unweighted descriptive statistics.

	Mean/Proportion	S.D.	Min.	Max.
Dependent variable				
*Mobile Phone Dependency*	2.38	.68	1	4
Level-1 (N = 1,788)				
*(W3) MPD*	2.41	.72	1	4
*Depression*	1.89	.56	1	3.9
*Female*	50%	___	0	1
*Academic satisfaction*	2.26	.74	1	4
*Delinquency*	.30	.73	0	6
*Aggressiveness*	1.96	.53	1	3.83
*Social interaction*	.89	.69	0	2.56
*Household income*	8.30	.58	5.19	10.60
*Both parents*	88%	___	0	1
*Parental abuse*	1.67	.61	1	4
Level-2 (N = 85)				
*Collective efficacy*	2.48	.22	1.42	3.50
*Aged population*	95.26	58.72	30.80	297.70
*Divorce rate*	2.23	.35	1.50	3.20
*College educated*	20.98	8.48	6.60	56.00
*Average income*	8.29	.34	6.86	9.65
*L2_Parental abuse*	1.66	.20	1.13	2.17

*Data source*: Korean Children and Youth Panel Survey (2012 & 2013)

### Analytical approach

KCYPS contains nested or clustered data due to the multistage stratified sampling design, which creates the possibility of non-independence of observations (adolescent respondents) across the contextual units (neighborhood clusters). Estimating conventional statistical methods such as Ordinary Least Squares (OLS) would thus lead to underestimation of standard errors, thereby increasing the probability of committing Type I error [70,71,72,73]. To remedy this problem, and, more importantly, to estimate direct and indirect (moderating) effects of neighborhood qualities on adolescent MPD, we fitted a series of multilevel (2-level) models using the latest version of HLM [74], the statistical software specifically designed to analyze hierarchically nested data. Following a standard procedure to avoid the collinearity problem, all non-dichotomous individual-level and neighborhood-level variables are grand-mean centered. Our choice of the centering approach is based on the fact that using grand-mean centered variables produces the parameter estimates necessary to check for the neighborhood contextual effects, while adjusting for individual-level characteristics (i.e., compositional effects) [75]. The estimated multilevel regression models are expressed as follows.

Individual-level model:

$$\begin{array}{ll} Y_{ij} & =\beta_{0j}+\beta_{1j}(Female_{i})+\beta_{2j}(Academic\;satisfaction_{i})+\beta_{3j}(Delinquency_{i})\\ & +\beta_{4j}(Aggressiveness_{i})+\beta_{5j}(Social\;interaction_{i})+\beta_{6j}(Household\;income_{i})\\ & +\beta_{7j}(Both\;parents_{i})+\beta_{8j}(Parental\;abuse_{i})+\beta_{9j}(W3\;MPD_{i})+\beta_{10j}(Depression_{i})+r_{ij} \end{array}$$

where *Y*_*ij*_ is the level of mobile phone dependency for respondent *i* in residential cluster *j*, β_0j_ represents the intercept, *β*_*ij*s_ are the parameter estimates, and *r*_*ij*_ is the individual-level random error term.

Neighborhood-level model:

$$\begin{array}{ll} \beta_{0j} & =\;\gamma_{00}+\gamma_{01}\left(L2\_Parental\;abuse_{j}\right)+\gamma_{02}\left(Collective\;efficacy_{j}\right)+\gamma_{03}\left(Divorce\;rate_{j}\right)\\ & +\gamma_{04}\left(Average\;income_{j}\right)+\gamma_{05}\left(College\;educated_{j}\right)+\gamma_{06}\left(Aged\;population_{j}\right)+u_{0j} \end{array}$$

$$\beta_{1j}=\gamma_{10},\;\beta_{2j}=\gamma_{20},\beta_{3j}=\gamma_{30}\ldots \beta_{8j}=\gamma_{80},\beta_{9j}=\gamma_{90},\beta_{10j}=\gamma_{100}$$

where *γ*_*00*_ is the intercept, *γ*_*01*_, *γ*_*02*_,… γ_06_ are the coefficients for the effects of six covariates on adolescent mobile phone dependency, and *u*_*0j*_ is the residential-level error term.

Cross-level moderating effects model:

$$\begin{array}{ll} \beta_{8j} & =\;\gamma_{80}+\gamma_{81}\left(L2\_Parental\;abuse\right)+\gamma_{82}\left(Collective\;efficacy\right)+\gamma_{83}\left(Divorce\;rate\right)\\ & +\gamma_{84}\left(Average\;income\right)+\gamma_{85}\left(College\;educated\right)+\gamma_{86}\left(Aged\;population\right)+u_{8j} \end{array}$$

## Results

Table 3 displays the results from multilevel analysis. Model 1 is the null model without any of the covariates. This model is run initially, akin to estimating a random effects one-way analysis of variance, to test whether or not running HLM is statistically necessary. The between-residential cluster variance component (τ = .02, χ^2^ = 171.02, df = 85, p < .001) suggests that there is significant variability in MPD. The intraclass correlation (ICC) shows that slightly over four percent of the variance in MPD among Korean youths is due to contextual-level effects. These results strongly indicate clustering of data and justify the need to use hierarchical linear modeling. Since the variables were group mean-centered, the intercept of 2.38 refers to the MPD score for an individual whose value for *Parental abuse* is equal to that of the neighborhood mean. Model 2 contains findings with all the (time-lagged) covariates, excluding the baseline dependency measure from the previous wave. Except for the two family background variables (*Household income* and *Both parents*), all of them emerge as significant predictors of the Korean adolescents’ MPD. Largely consistent with prior research, girls exhibit higher levels of dependency than do boys (p < .001), those who are more satisfied with their academic achievements are less dependent (p < .01), and individuals who spend more time socializing with friends show more dependency (p < .001). Delinquent behaviors (p < .001) and aggressive nature (p < .001) are also positively associated with adolescent MPD. Having reported depressed symptoms from the previous year leads to heightened dependency as well (p < .01). Finally, for the main purposes of this study, we find that, net of these controls, children who describe their parents as being abusive score significantly higher on the self-identified MPD scale. For each unit increase in *Parental abuse*, the corresponding composite score rises by 0.18 (p < .001).

**Table 3:** Hierarchical linear models estimating the association between parental abuse and mobile phone dependenc*y* (KCYPS 2012 & 2013).

	Model 1 Coef (SE)	Model 2 Coef (SE)	Model 3 Coef (SE)	Model 4 Coef (SE)	Model 5 Coef (SE)
Constant	2.38 (.026)***	2.32 (.058)***	2.36 (.053)***	2.35 (.053)***	2.35 (.053)***
Individual-level (N = 1,788)					
*Female*		0.27 (.038)***	0.15 (.035)***	0.16 (.036)***	0.16 (.035)***
*Academic satisfaction*		-0.05 (.022)*	-0.03 (.020)^#^	-0.04 (.020)^#^	-0.04 (.020)*
*Delinquency*		0.10 (.024)***	0.08 (.022)***	0.08 (.022)***	0.08 (.022)***
*Aggressiveness*		0.25 (.035)***	0.17 (.032)***	0.17 (.032)***	0.16 (.032)***
*Social interaction*		0.12 (.024)***	0.06 (.022)**	0.07 (.022)**	0.06 (.022)**
*Household income*		0.02 (.034)	-0.03 (.031)	-0.03 (.032)	-0.03 (.032)
*Both parents*		-0.07 (.056)	-0.05 (.051)	-0.05 (.051)	-0.05 (.051)
*Depression*		0.08 (.029)**	-0.01 (.027)	-0.01 (.027)	-0.01 (.027)
*Parental abuse*		0.18 (.028)***	0.15 (.026)***	0.15 (.026)***	0.15 (.032)***
*(W3) MPD*			0.41 (.023)***	0.41 (.023)***	0.42 (.023)***
Neighborhood-level (N = 85)					
*Collective efficacy*				0.04 (.164)	0.04 (.156)
*Aged population*				0.00 (.001)	0.00 (.001)
*Divorce rate*				0.09 (.080)	0.07 (.075)
*College educated*				0.00 (.004)	0.00 (.004)
*Average income*				0.12 (.099)	0.08 (.094)
*L2_Parental abuse*				0.18 (.134)	0.21 (.129)
Variance component (L-1)	.445	.362	.292	.292	.286
Variance component (L-2)	.020***	.017***	.017***	.015***	.015***
Deviance	3970.75	3413.29	2936.89	2931.17	2917.19

*Note*: Parameter estimates are from unit-specific models. Data have been adjusted using person weights to account for nonresponse and probability of selection.

^#^ p < .1,

* p < .05,

** p < .01,

*** p < .001 (two-tailed tests)

For stricter testing of the causal association between abusive parenting style and MPD, we introduce the baseline dependency measure from the earlier wave, *(W3) MPD*, in Model 3, which is significantly related to the outcome measure (p < .001). Inclusion of this variable, for the most part, does not substantively alter the strengths or magnitudes of other variables. One noticeable exception is *Depression*, which falls below the conventional level of significance (p-value of 0.05). In other words, when we hold constant the children’s respective level of MPD from the previous year, the impact of depression disappears, suggesting that the depression-MPD linkage is powerfully mediated by the baseline dependency (i.e., more depressed kids are much more prone to problematic usage of mobile phone). The key finding here is that, even after we control for the baseline measure, the association between parental abuse and MPD remains robust (p < .001), though the effect size decreases slightly to 0.15.

Having estimated individual-level models, we now move to contextual-level covariates. Model 4 shows results from incorporating the six neighborhood-level variables, none of which turns out to be significant predictors. In models not shown, we group-mean centered L-1 variables to examine possible aggregate or collective level association between Yj and Xj. According to those results, the coefficients for *Divorce rate* and *L2_Parental* reach the level of significance. However, only under grand-mean centering, the intercept variance represents between-group variance in the outcome variable adjusted for L-1 predictors. Thus, as indicated by Model 4, when individual-level variables are taken into account through grand-mean centering, these two neighbor-level variables become insignificant, indicating that there is no contextual effect net of compositional effects. One powerful feature of the multilevel approach is that it allows for the slopes for individual-level variables to vary across the contextual (neighborhood) unit. That is, for example, we can examine if the influence of parental abuse on MPD shifts from one residential context to another. Model 5 is estimated to validate this possibility. Results indicate that the parental abuse-MPD nexus is in fact not constant but fluctuates across different residential neighborhoods throughout Korea, as indicated by the parameter estimate for *Parental abuse* (p < .001) and the variance component for its slope (*u*_*8*_ = 0.017, χ^2^ = 112.32, df = 82, p < .05).

Findings in Table 3 provide evidence that there is indeed a positive and robust relationship between abusive parenting style and MPD in Korea, while controlling for measures of neighborhood quality. Having demonstrated this, we now turn to the possible interactive, or moderating, effects of neighborhood variables in predicting adolescent MPD. Statistical results from running cross-level interaction models are summarized in Table 4. Out of six possibilities, two emerge as significant, namely those involving the average educational attainment level of residents (*College educated*) and the proportion of elderly residents (*Aged population*). On the one hand, according to Model 5, the association between parental abuse and MPD is weaker (p < .05) in a residential setting characterized by higher proportion of college graduates. On the other hand, as Model 6 reveals, this association is stronger (p < .05) in more aged communities, those with more residents who are 65 years of age and over.

**Table 4:** Cross-level interaction effects between neighborhood characteristics and parental abuse on mobile phone dependency (KCYPS 2012 & 2013).

	Model 1	Model 2	Model 3	Model 4	Model 5	Model 6
	Coef. (SE)	Coef. (SE)	Coef. (SE)	Coef. (SE)	Coef. (SE)	Coef. (SE)
(Neighborhood-level)						
*L2_Parental abuse*	0.18 (.144)	0.17 (.144)	0.18 (.144)	0.18 (.144)	0.21 (.128)	0.20 (.129)
*Collective efficacy*	-0.04 (.176)	0.01 (.180)	-0.03 (.176)	-0.03 (.176)	0.05 (.156)	0.04 (.156)
*Divorce rate*	0.13 (.085)	0.13 (.085)	0.14 (.085)	0.13 (.085)	0.08 (.075)	0.07 (.075)
*Average income*	0.17 (.105)	0.16 (.105)	0.16 (.105)	0.16 (.107)	0.08 (.094)	0.08 (.094)
*College educated*	0.00 (.004)	0.00 (.004)	0.00 (.004)	0.00 (.004)	0.00 (.004)	0.00 (.004)
*Aged population*	0.00 (.001)	0.00 (.001)	0.00 (.001)	0.00 (.001)	0.00 (.001)	0.00 (.001)
(Cross-level interactions)						
*L2_Parental abuse* × *Parental abuse*	0.09 (.204)					
*Collective efficacy* × *Parental abuse*		0.32 (.256)				
*Divorce rate* × *Parental abuse*			0.12 (.092)			
*Average income* × *Parental abuse*				-0.13 (.140)		
*College educated* × *Parental abuse*					-0.01 (.004)*	
*Aged population* × *Parental abuse*						0.00 (.001)*
Variance component (L-1)	.354	.354	.354	.354	.285	.286
Variance component (L-2)	.016***	.015***	.015***	.016***	.014***	.014***
Deviance	3400.56	3398.78	3398.66	3399.66	2910.06	2911.83

*Note*: Parameter estimates are from unit-specific models. Data have been adjusted using person weights to account for nonresponse and probability of selection. Above models control for all the individual-level covariates (including *Parental abuse*) shown in Table 3.

^#^ p < .1,

* p < .05,

** p < .01,

*** p < .001 (two-tailed tests)

**Fig 2:**
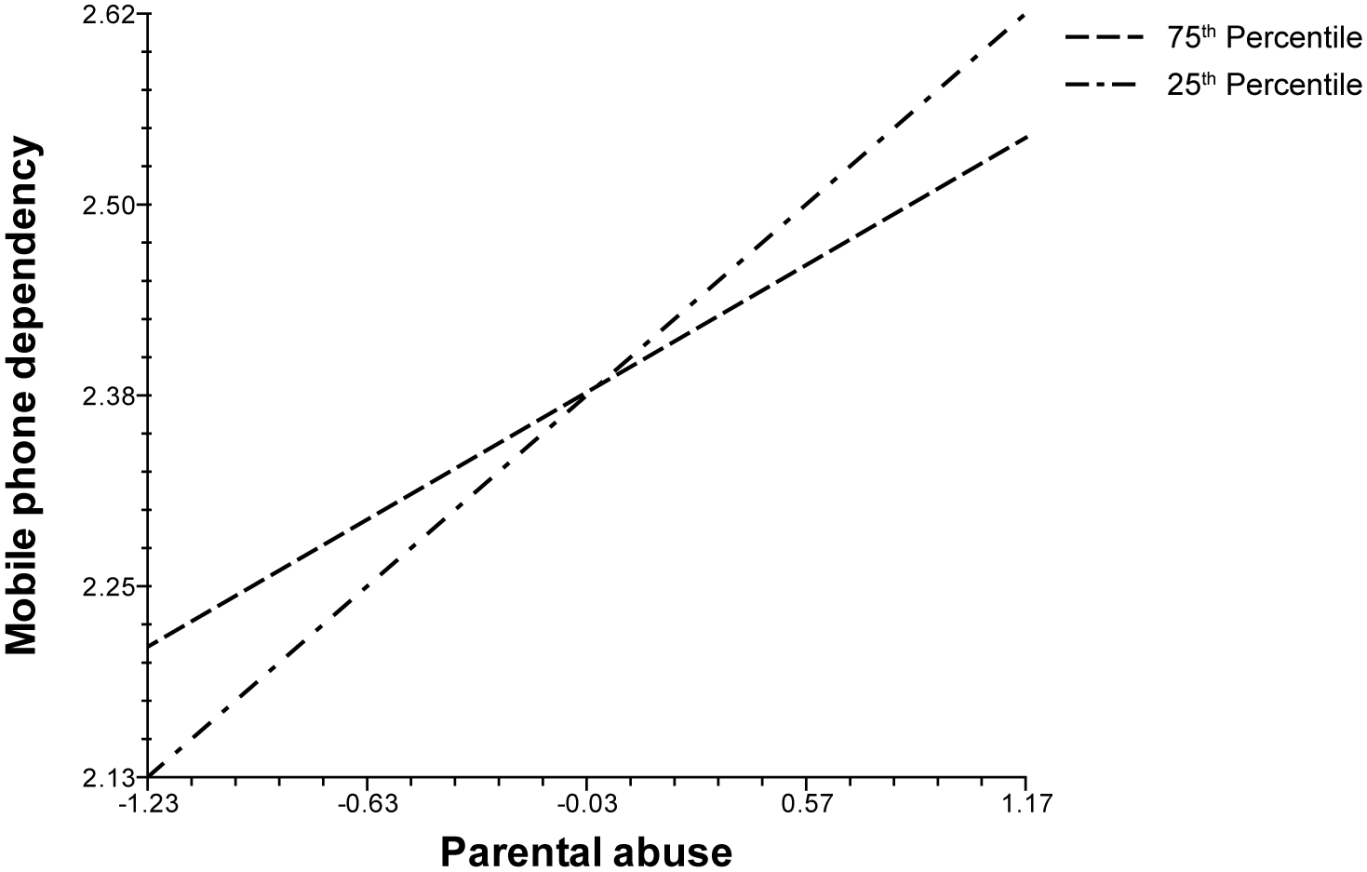
Cross-level interaction with mean education.

**Fig 3:**
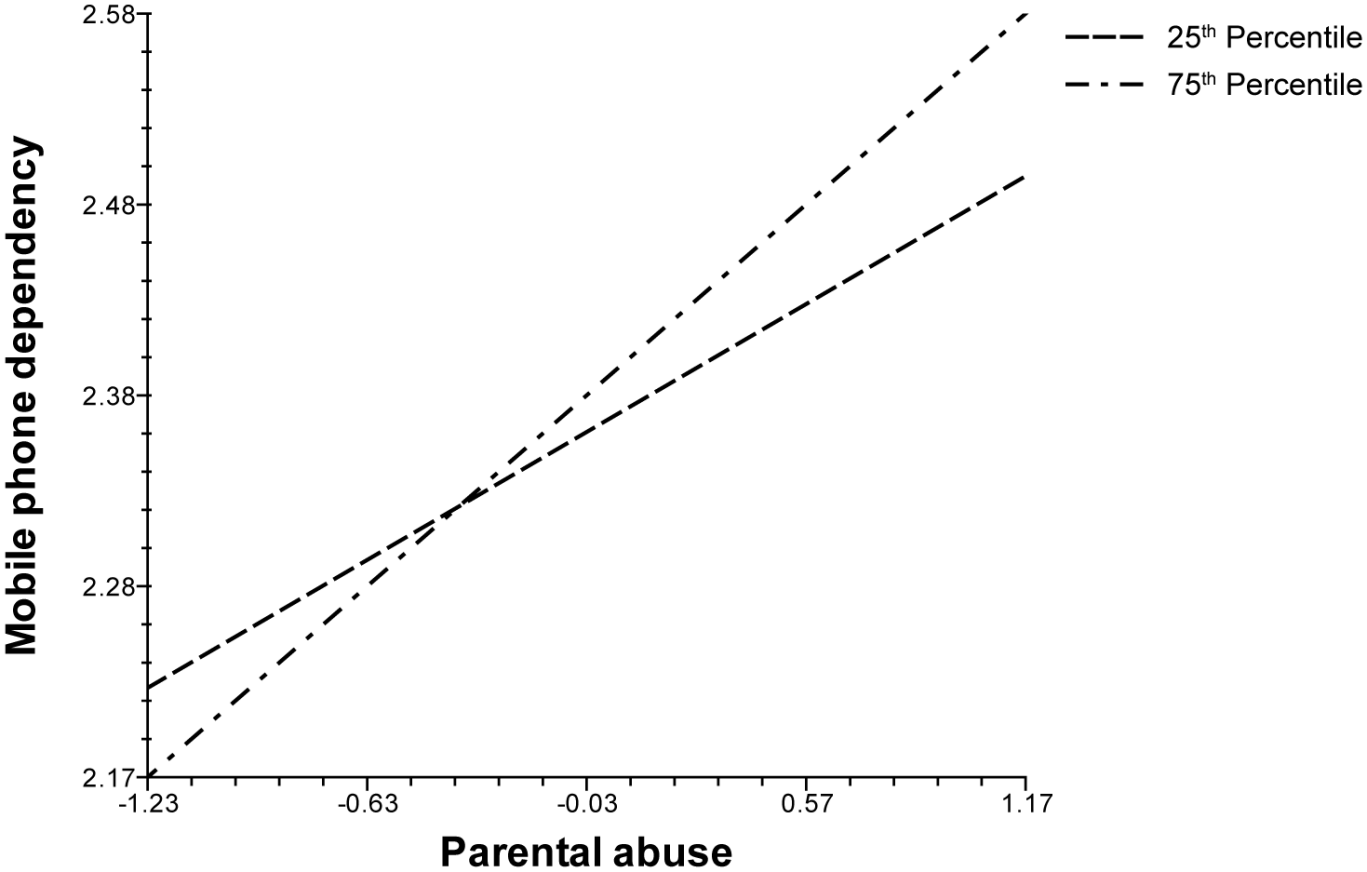
Cross-level interaction with mean age.

As for interpretation, we conjecture that neighborhoods with better educated individuals possess functional qualities and facilities that can cushion the negative impact of parental abuse on adolescent behaviors and development, such as MPD. This should not be interpreted to mean, however, that higher educated parents necessarily possess more knowledge and better skills in rearing children. Making such an interpretation is equivalent to committing ecological fallacy by wrongly drawing micro-level conclusions based on aggregate data. What we can infer nevertheless is that at the aggregate level, there is a relationship between neighborhood quality (i.e., mean educational level) and *average* MPD. It is possible that communities consisting of more college graduates, for example, are in a more advantaged position, or have the collective capacity, to facilitate the well-being of youths and to protect them from harm. In other words, such communities can better buffer the negative effects of adolescent development. Hence, although a child who experiences parental abuse is more likely to become a problematic mobile phone user, this tendency is lessened in a higher quality neighborhood, as conceptualized in terms of average educational attainment. The opposite would be true in community settings with more elderly residents, since old age is typically associated with poorer quality neighborhoods with limited resources in Korea [76,77]. Older communities may also be ineffective in playing the buffering role since elderly residents are less physically mobile and have relatively limited social interaction. Therefore, the negative result of parental abuse in the form of MPD would be more reinforced in aged communities. These contingent relationships are graphically illustrated in Figs 2 and 3, for *College educated* and *Aged population*, respectively. Both figures highlight their moderating effects on the parental abuse-MPD linkage at 25^th^ and 75^th^ percentile values (i.e., “relatively uneducated” vs. “relatively educated” and “very young” vs. “very old”), while holding all other covariates in the model at their numerical means.

## Discussion

With increasing development and decreasing costs of communications devices, mobile phone ownership has become a dominant form of technology adoption in contemporary societies the world over. At the same time, MPD has surfaced as a widespread social and public health problem, prompting concerned observers to speak of “smartphone addiction” especially among the younger generation [78,79]. Clearly, Korea has not been an exception to this trend. Both in the media and in academic writings, much has been said about the inevitable popularization of mobile phones among Korean children and related psychological dependency and dysfunctional behaviors [80]. A growing volume of research on this very topic attests to the fact that contemporary Korean society faces a novel epidemic of the digital era, as its youth turn into “mindless slaves” to mobile devices [81,82].

While a great deal of efforts went into investigating the connection between MPD and concomitant internalizing problems, we know relatively little about the “social” factors (e.g., quality of parent-child relationship or parental abuse) that precede it. Due to the cross-sectional nature of data analyzed, previous studies are also largely confined to demonstrating correlations between psychological, mental and behavioral traits and MPD. More often than not, many of those studies rely on convenient or non-probability sampling method, making it impossible to make claims about the general population. Furthermore, multilevel models have rarely been utilized to probe independent and interactive effects of contextual-level factors such as neighborhood characteristics. Investigating this topic has both methodological and theoretical merits. Methodologically, reverse causation does not pose a serious problem. A kid whose parents are abusive may exhibit higher levels of MPD (as a way of escape, for example). The reverse scenario, however, is much less likely, i.e., that a child’s dependency leads to abusive parenting. In other words, we are able to present a more “causal” story here, supplemented by the use of data over time that properly sets the temporal order between the variables of interest. In this study, we discovered new findings that have relevant implications for not only Korea, a country with one of the highest smartphone users in the world, but also other countries similarly experiencing the rise of adolescent MPD.

To recap, we find strong support for the claim that parental abuse adds to children’s addictive behaviors and attitudes concerning the mobile phone use, net of controls (including the dependency measure from the previous year) at individual and neighborhood levels. Being surrounded by peers in the same neighborhood who experience greater parental abuse is not related to one’s own MPD, however, after adjusting for individual-level variables. In addition, the parent abuse-MPD association is not static but varies according to two neighborhood features: average educational level and age composition of residents. Specifically, a neighborhood with more college-educated inhabitants buffers the impact of parental abuse on MPD, while another with more elderly inhabitants exacerbates the effect. There is an important caveat here in interpreting these results. The contingent relationships we found underscore “residential” characteristics, not the characteristics of “residents.” Certain neighborhoods are equipped with the right built environment, supportive community culture, and ample social resources that can protect children from the stressors (parental abuse) of externalizing behaviors (mobile phone dependency). Neighborhoods with relatively younger and better educated residents may be more likely to possess such capacity or advantage. This tendency, however, in no way denies the possibility that older and/or less educated individuals are less capable of providing a buffering mechanism to youths. The two related yet distinct levels of analysis- collective and individual- should not be conflated.

On a theoretical note, there is a rich literature on parent-child relationship as a protective factor against delinquency [30,83,84]. Studies show that children who are better monitored and supervised by their parents are less delinquent, ceteris paribus. Much of this scholarship highlights the positive, or functional, role of parenting couched in the language of “intergenerational closure” [85] or social capital [86]. Parent-child relationship, however, is a double-edge sword. That is, it can also be a risk factor, as this study has shown in relation to the increasingly global phenomenon of MPD among the youth. Future research should take into account the conditions under which different types of parenting can aid, as well as hinder, mental and physical development of children.

## Conclusions

That we found two moderating effects of neighborhood-level characteristics on the parental abuse-MPD nexus has major implications for the future well-being of adolescents in the age of Internet-based hyperconnectivity. One of the hallmarks of current population trends in the world is rapid ageing. According to a report by World Health Organization [87], humanity is on the verge of “a demographic milestone”: Before 2020, for the first time in recorded human history, the number of older individuals (65 years and older) will outnumber the young (under 5 years of age). In just about every society, younger members will thus live in the midst of an ever-increasing number of older adults. We also know that the elderly population is not evenly distributed geographically within and across countries. Rather, pockets of residential neighborhoods and communities exist with disproportionately high or low numbers of older inhabitants. The geocoded data drawn from official government statistics, which were included in our analysis, clearly suggest this to be true for Korea. And we imagine this pattern to hold in other countries as well in varying degree. What this suggests is that globally adolescents face and will continue to face unequal levels of exposure to elderly co-residents, which, according to our findings, has significant consequences in terms of MPD.

Decision concerning where to live is largely a financial one. A person cannot simply move to a particular neighborhood without careful considerations of the housing cost, among other sources of expenditure. In other words, household income plays a critical part, which in capitalist economies is primarily determined by the would-be mover’s educational level. Income and years of schooling in fact go hand-in-hand everywhere, irrespective of geography, culture and, to lesser extent, politics. If so, we can envision the following scenario. Children are born to parents of unequal means. Some parents are better educated than others and thus able to choose higher-quality neighborhoods, which are often characterized by lower percentage of older residents, as more financially able retirees may opt to move out and into institutionalized settings such as private retirement homes. A community disproportionately populated by elderly members who have been “left behind”, then, can signal residential unattractiveness in term of, for example, poor-quality schools which better-educated parents tend to shun. This thought experiment leads to a rather socially bleak conclusion: Household inequality will reinforce the negative impact of parental abuse on children’s dependency on mobile phones and, most likely, other networking or gaming devices.

The status quo and the outlook delineated above call for effective policy intervention. Residential integration based on age and education seems to be the biggest and the most challenging issue at hand, since they are critical drivers of community infrastructure [59]. To move toward an equitable demographic makeup of neighborhoods, governments should endeavor to create better schools in low-income communities by channeling material resources and recruiting good teachers. They should also invest in health-promoting facilities and programs for aging populations to prevent the outward flight of more privileged members. Last but not least, public assistance (in the form of after-school activities, etc.) is needed for adolescents, especially those in abusive families, to help control and reduce their dependency on mobile devices. This to-do list would be considered a herculean task for any government in power. Nevertheless, the accelerating speed of population ageing, coupled with the widening income gap, in most countries does not bode well for the psychological health of younger generations. And postponing intervention, unfortunately, would only raise its costs in the end.
